# The evaluation of e-learning resources as an adjunct to otolaryngology teaching: a pilot study

**DOI:** 10.1186/s12909-019-1618-7

**Published:** 2019-06-03

**Authors:** Ronald Yoon-Kong Chin, Richard Tjahjono, Michael John Raymond Rutledge, Tim Lambert, Nathaniel Deboever

**Affiliations:** 1 Department of Otolaryngology Head and Neck Surgery, Nepean Hospital, Derby Street, KINGSWOOD, Sydney, NSW 2747 Australia; 2 Faculty of Medicine, University of Sydney, Sydney, Australia; 3 Faculty of Medicine, Western Sydney University, Sydney, Australia

**Keywords:** Otolaryngology education, E-learning, Clinical examination, Medical curriculum, Medical student

## Abstract

**Background:**

The concept of e-Learning has been rapidly accepted as an important component of medical education and is especially adept at teaching clinical skills. However, their impact on learning, particularly in Otolaryngology Head and Neck Surgery (OHNS) medical school curriculum, has yet to be adequately explored. The aim of this pilot study is to develop interactive e-Learning resources and evaluate their impact in enhancing OHNS teaching in medical school.

**Methods:**

This pilot study is a randomized controlled trial assessing the effectiveness of e-Learning resources in enhancing the current traditional lecture and tutorial-based teaching of OHNS in medical school. Nineteen final-year medical students from the University of Sydney were recruited for this study, who were randomly allocated into intervention group with additional e-Learning resources (Group A) and control group (Group B). Student knowledge was assessed through objective structured clinical examinations (OSCE) with use of standardized forms for objective scoring. Assessors were blinded to student randomization status. A post-study questionnaire was distributed to assess student feedback on the e-Learning resources.

**Results:**

Eight students were allocated to Group A and 11 students to Group B. Group A performed significantly better than Group B in the overall examination scores (78.50 ± 13.88 v. 55.82 ± 8.23; *P* = < 0.01). With the minimum pass mark of 65%, the majority of students in Group A was able to pass the OSCE assessments, while the majority of students in Group B failed (87.50% v. 9.10%; *P* = 0.01). The post-test questionnaire on the e-Learning resources showed very favorable feedback from the students’ perspective.

**Conclusion:**

Results from our pilot study suggests that the use of interactive online e-Learning resources can be a valuable adjunct in supplementing OHNS teaching in medical school, as they are readily accessible and allow flexible on-demand learning. Future studies involving large numbers of medical students are needed to validate these results.

## Background

Over the past few years, the concept of e-Learning has been rapidly accepted as an important component of medical education [1]. The definition of e-Learning is varied; however in its most rudimentary form, e-Learning is a method that utilizes internet-based resources for teaching and learning purposes. In surgery, e-Learning is not seen as a single entity but rather a combination of teaching methods, such as online lectures, tutorials or virtual case studies [2]. The literature has described a number of advantages of e-Learning including; (1) ease of access, (2) increased flexibility of student learning, (3) increased interactivity between educators and students, (4) decreased content review times and (5) opportunity for immediate self-assessment [3–5].

E-Learning resources have a broad range of uses in medical education and they are especially adept at teaching clinical skills. Clinical skills require teaching of multiple domains, including declarative knowledge (underlying facts), procedural steps (the “how” of performing a task) and clinical reasoning [6]. Whilst we understand that repetition and feedback supports better competence in clinicians, it has been shown that multimedia and e-Learning tools have improved testing in both declarative knowledge and procedural step domains [7, 8].

Clinical presentations related to otolaryngology head and neck surgery (OHNS) are common, comprising of > 20% of all presentations in the primary care setting [9–11]. However, studies have reported poor basic knowledge and exposure to OHNS in the medical school and primary care settings, demonstrating a mismatch between this educational need and the current medical curriculum [12–14]. This mismatch may contribute to diagnostic errors, which account for approximately 14% of negligent adverse events in hospitalized patients [15, 16].

As new technological developments rapidly emerge within medical education, e-Learning has the potential to be an invaluable adjunct to traditional OHNS teaching. Therefore, the aim of this pilot study is to evaluate the educational impact of interactive e-Learning resources in enhancing OHNS teaching through a small representative group of medical students in regard to knowledge acquisition and application.

## Methods

This pilot study is a randomized controlled trial assessing the effectiveness of e-Learning resources in enhancing the current traditional lecture and tutorial-based teaching of OHNS in medical school. Participants were randomized according to the CONSORT statement recommendations. Ethics approval was granted by the University of Sydney Human Research Ethics Committee.

Final-year medical students from the University of Sydney at the clinical school where the study was conducted were recruited to participate in this study between July to September 2017. This was the student group of choice for selection in the study as they have completed all standard training in OHNS as per the current University of Sydney curriculum within a similar timeframe. Recruitment was done through broadcast emails, flyers and lecture announcements. All participating students were provided with an information sheet and gave written informed consent. To supplement the teaching syllabus, interactive e-Learning teaching resources were designed by the authors, which focused on the OHNS clinical examination skills that had been previously taught to the students via traditional methods.

Materials in the interactive e-Learning teaching resources cover existing topics from the University of Sydney medical school curriculum, and included videos of OHNS clinical examinations (thyroid, rhinological, otological, oral and head & neck examinations), lasting approximately 26 min. Layered information and interactive questions were placed at intervals throughout the video resources, where students can decide to interact as they see fit. A quiz at the end of each video resource is also available for the purposes of self-assessment and better knowledge retention. The clinical examination techniques demonstrated in the video serve as a teaching guide, not a benchmark in which students are assessed on. In addition, no limitations were imposed on the use of these e-Learning resources, although usage of the e-Learning resources were not logged due to system limitations. The students’ ability to demonstrate OHNS clinical examination skills and knowledge was assessed through objective structured clinical examinations (OSCE), which is a standard examination format in the University of Sydney medical school.

**Fig. 1:**
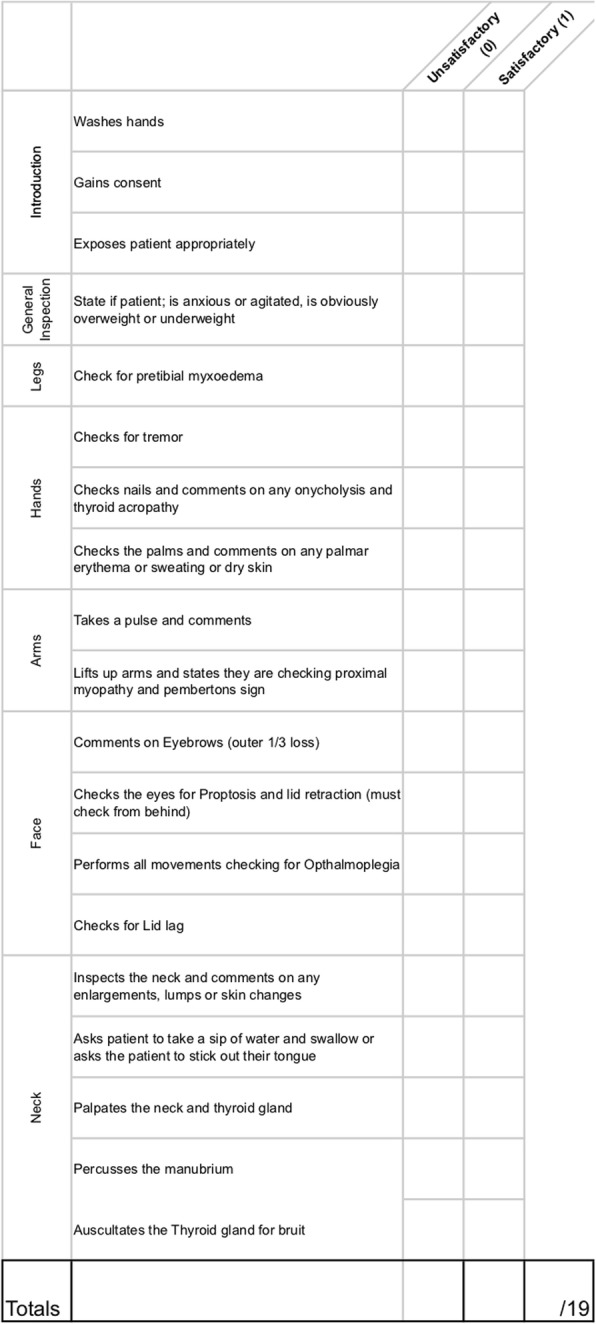
Thyroid Examination Marking Sheet

**Fig. 2:**
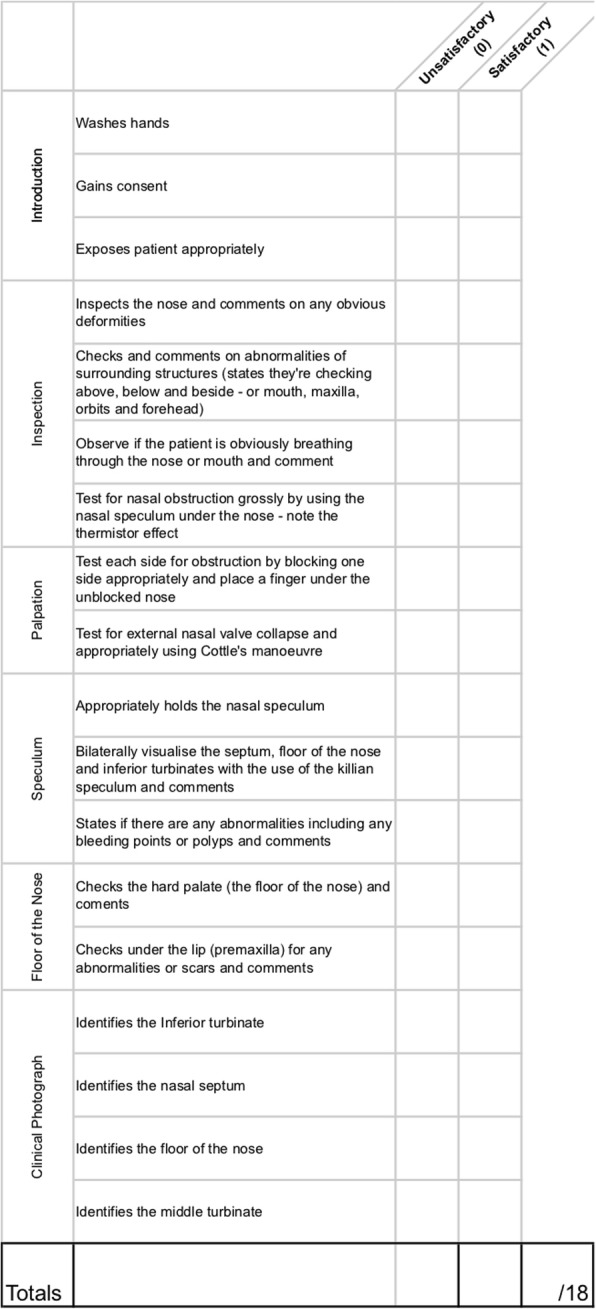
Rhinological Examination Marking Sheet

**Fig. 3:**
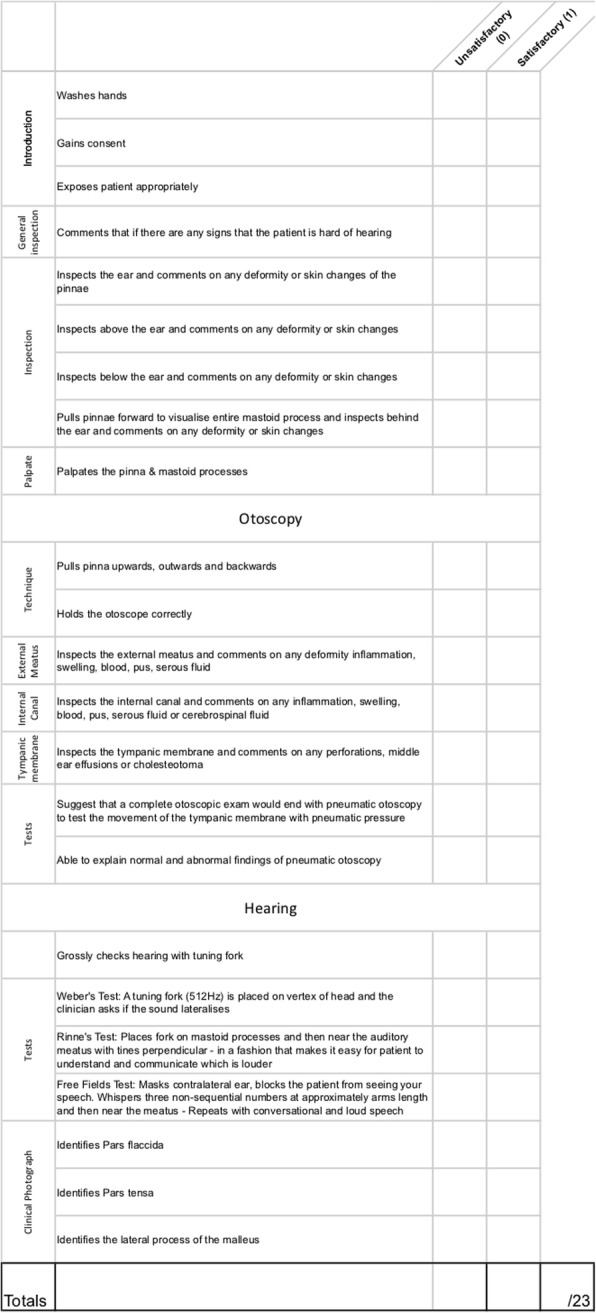
Otological Examination Marking Sheet

**Fig. 4:**
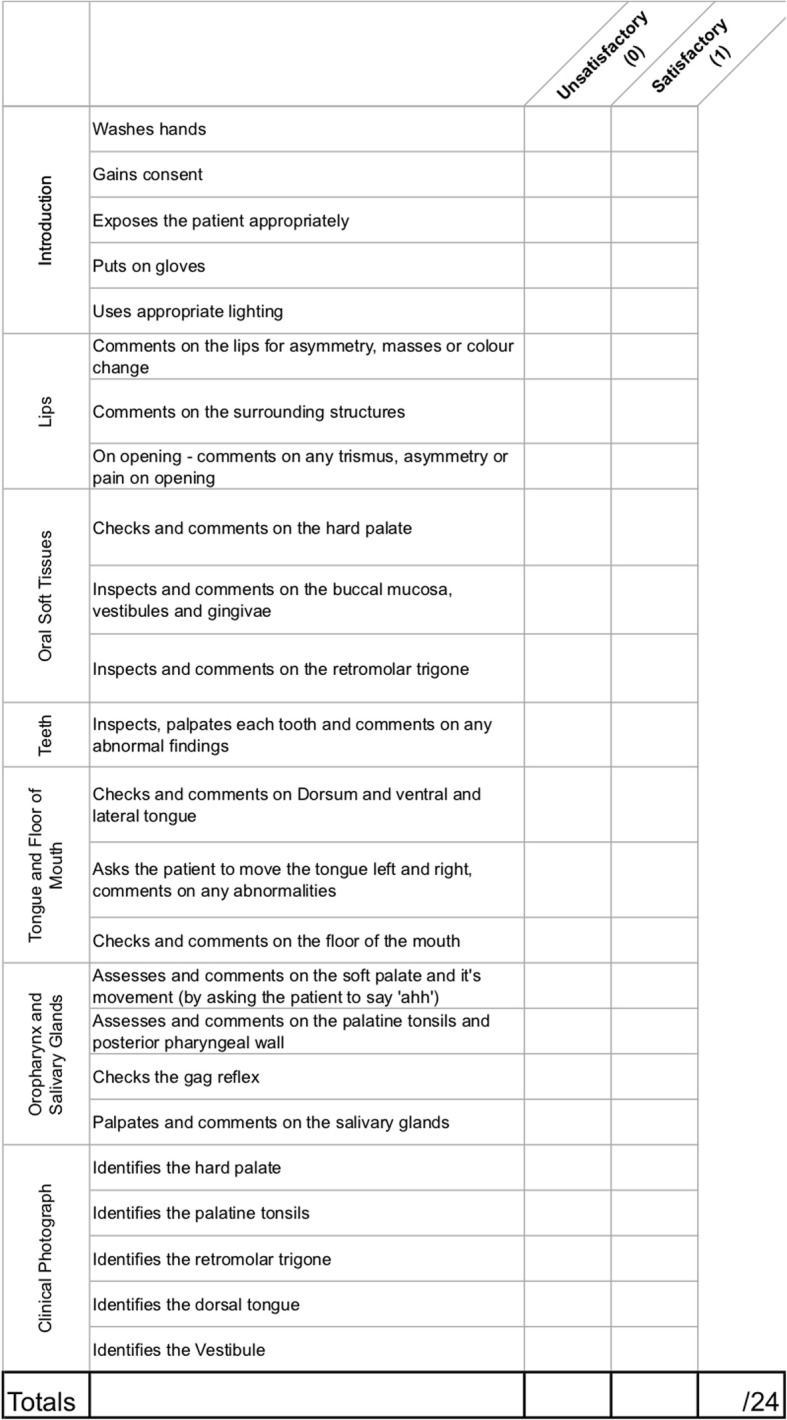
Oral Examination Marking Sheet

**Fig. 5:**
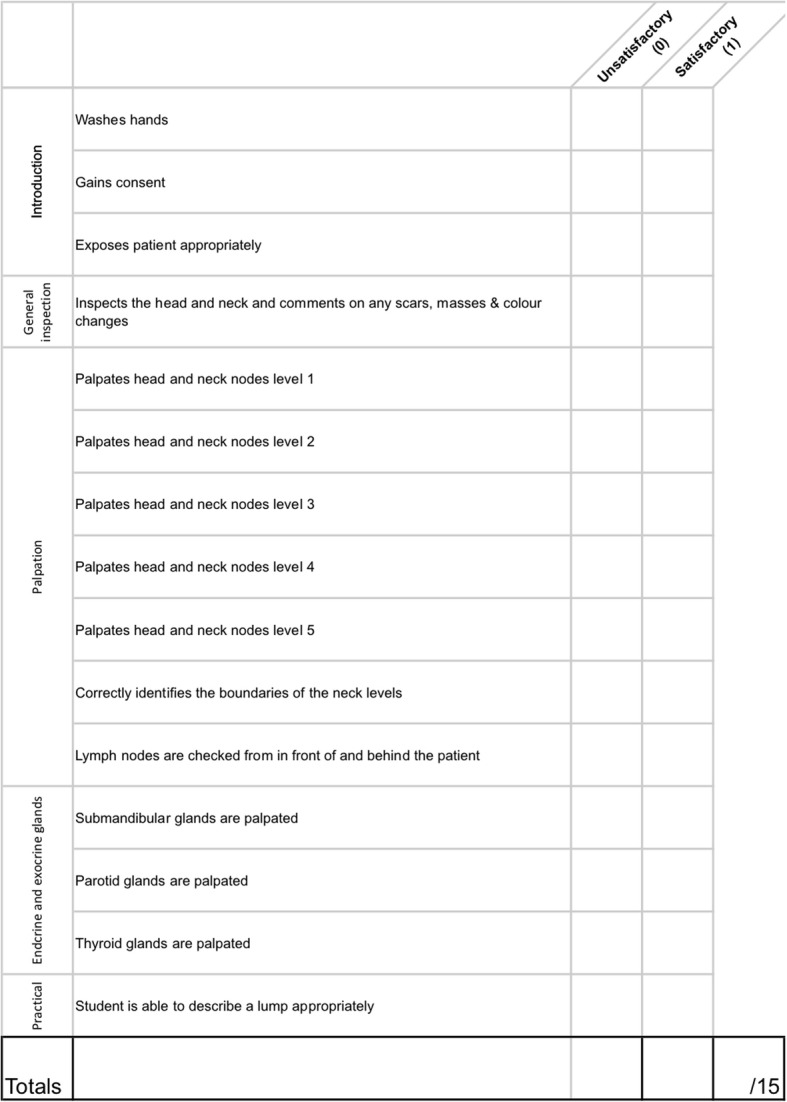
Head and Neck Examination Marking Sheet

Prior to formal OSCE assessment, students were randomized into two groups. A randomization technique was done with the use of a computer random number generator. Each student was assigned a number, which was used to determine group allocations. Students in the intervention group were provided access to the e-Learning teaching resources through a unique link and login details in addition to self-directed study (Group A). Students in the control group did not have access to the e-Learning teaching resources and were advised to use “study as usual” (Group B). Each student was assessed on thyroid, rhinological, otological, oral, and head & neck clinical examination skills. These were divided into five 10-min stations. Students were given 8 min to examine a volunteer surrogate patient and then had 2 min of question time for each station. Evaluation was done through standardized assessment forms using an analytical scoring rubric, which were developed by the authors (Figs. 1, 2, 3, 4, 5). Using this method minimizes examiner bias because students either performed the action, or did not perform the action. This was upon recognition that there are many valid techniques in performing a full otolaryngologic examination; emphasis was placed upon the student’s ability in elucidating clinical signs to enable accurate diagnosis of the relevant pathology.

Six physicians who were affiliated with the University of Sydney Faculty of Medicine participated in the study as examiners, who received instructions prior to student assessment in order to minimize inter-rater differences in marking. To ensure the highest level of objectivity, examiners were not allowed to assess students that they have personally known from clinical rotations or small tutorial groups. The examiners were blinded to the two groups, and the number of students assessed was fairly distributed to all examiners. Two examiners were used to assess each student to ensure consistency of the scoring process. The examiners did not share scoring results with each other, and the final station score was an average of each examiner’s score. Overall and individual station scores were collected, with a minimum pass mark of 65%, which is the standard benchmark used by the University of Sydney medical school.

At the end of the study, all participating students were able to obtain direct feedback and given open access to the interactive e-Learning teaching resources for their own personal learning. In addition, the survey questionnaire was also distributed to every student following the release of OSCE results to provide feedback on the e-Learning resources, which comprises 10 questions. The survey was intended to obtain feedback on the quality, ease of access and practicability of the learning materials provided in the e-Learning resources to ensure ongoing student engagement and quality improvement process. Each question was scaled from 1 (strongly disagree) to 10 (strongly agree).

Statistical analysis was performed by the use of IBM SPSS statistics software version 25.0 (SPSS Inc., Chicago, Ill, United States). Results are presented as value ± standard deviation. Student examination scores between the two groups were compared using an independent t-test. Differences between students who passed the clinical examination stations were compared using a chi-squared test. A *P*-value of < 0.05 is considered statistically significant, with 95% confidence intervals.

## Results

Nineteen final-year medical students took part in the study (out of 42 in the clinical school where this study was conducted). Eight students were allocated into Group A and the remaining 11 students were allocated into Group B. The unequal distribution of students was a result of the randomization process. Average scores for Group A and B are described in Table 1. Students in Group A were able to perform significantly better than Group B in the overall (78.50 ± 13.88 v. 55.82 ± 8.23; *P* = < 0.01) and individual station scores. Lower individual station scores were seen in the rhinological (76.25 ± 19.66 v. 42.91 ± 11.17; *P* = < 0.01) and oral (69.25 ± 22.31 v. 51.91 ± 11.36; *P* = 0.04) examination stations for both groups.

**Table 1:** Average examination scores between Group A and Group B; independent t-test was used for statistical analysis

	Group A (*n* = 8)	Group B (*n* = 11)	*p*-value
Overall (%)	78.50 ± 13.88	55.82 ± 8.23	< 0.01
Thyroid (%)	88.13 ± 10.62	62.91 ± 14.27	< 0.01
Rhinological (%)	76.25 ± 19.66	42.91 ± 11.17	< 0.01
Otological (%)	79.00 ± 15.73	62.82 ± 14.68	0.03
Oral (%)	69.25 ± 22.31	51.91 ± 11.36	0.04
Head and Neck (%)	81.63 ± 11.72	57.00 ± 16.56	0.02

Pass rates for Group A and B are depicted in Table 2**.** With the minimum pass mark of 65%, the majority of students in Group A were able to pass the OSCE assessments, while the majority of students in Group B failed (87.50% v. 9.10%; *P* = 0.01). Every student in Group A passed the thyroid and head & neck examination stations, with satisfactory pass rates on the otological examination station (87.50%). Lower pass rates were seen on rhinological and oral examinations (62.50 and 62.50% respectively). In contrast, only half of the students in Group B passed the thyroid and head & neck examinations (54.55 and 54.55% respectively), with most failing the rhinological, otological and oral examinations (0, 36.36 and 9.10% respectively).

**Table 2:** Number of students who passed the examination in Group A and Group B; minimum pass mark of 65%; chi-square test was used for statistical analysis

	Group A (*n* = 8)	Group B (*n* = 11)	*p*-value
Overall (%)	7 (87.50%)	1 (9.10%)	0.01
Thyroid (%)	8 (100%)	6 (54.55%)	0.03
Rhinological (%)	5 (62.50%)	0 (0%)	0.02
Otological (%)	7 (87.50%)	4 (36.36%)	0.03
Oral (%)	5 (62.50%)	1 (9.10%)	0.01
Head and Neck (%)	8 (100%)	6 (54.55%)	0.03

In addition, all 19 participating students completed the post-study survey with a 100% response rate. Students provided favorable feedback on the e-Learning resources, with average scores of 8 or higher for each question **(**Table 3**)**.

**Table 3:** Post-study survey results on the e-Learning resources for feedback purposes; each question is rated from 1 (strongly disagree) to 10 (strongly agree)

Question	Average Score out of 10
Q1. Teaching material provided in the e-Learning resources was clear and easy to understand	8.84 ± 0.96
Q2. Teaching material provided in the e-Learning resources was delivered at an appropriate pace and in a logical sequence	8.37 ± 1.21
Q3. E-Learning resources were helpful in understanding basic OHNS concepts	9.26 ± 0.93
Q4. E-Learning resources were helpful in memorization and revision of OHNS clinical skills	8.63 ± 0.96
Q5. E-Learning resources were easy to use and navigate	8.37 ± 1.34
Q6. E-Learning resources were enjoyable resources to use for additional study	8.11 ± 1.10
Q7. E-Learning resources are useful tools in supplementing traditional OHNS teaching in medical school	9.47 ± 0.90
Q8. I would recommend these OHNS e-Learning resources to another student	8.57 ± 1.12
Q9. The OSCE assessment process in this study was fair	8.05 ± 0.97
Q10. I feel that I am able to perform basic OHNS examinations better as an junior doctor in the Emergency Department with the additional use of e-Learning resources	8.00 ± 0.88

## Discussion

The importance of OHNS knowledge in primary care has been well established. Otolaryngologic presentations such as oropharyngeal pain, epistaxis, rhinorrhea and otalgia are common in the primary care setting, accounting for over 20% of presenting complaints in the adult population and up to 50% in the pediatric population [9, 17, 18]. However, OHNS has been largely under-represented in the medical school teaching syllabus [19–22]. A study in 2004 surveying OHNS teaching in 27 medical schools in the United Kingdom revealed that 6 medical schools (22%) did not have mandatory OHNS clinical attachments. Fifty-eight percent of all OHNS attachments are combined with other specialties, with an average length of time spent in the clinical attachment of 1.5 weeks over 5 years [19]. Another study in the United Kingdom surveyed senior trainees in emergency medicine, where 75% of respondents felt that medical school OHNS teaching was inadequate [20]. Similarly, a lack of compulsory OHNS clinical attachments, limited length of clinical attachments and variability in teaching syllabus were also seen in Canadian medical schools [21].

At the University of Sydney medical school, students have mandatory lectures and tutorials in OHNS. Our additional online e-Learning resources are visualized as an adjunct to the OHNS teaching curriculum and is not designed to be a standalone teaching tool. We believe that these resources are utilized best when they are used in conjunction with term attachments, lectures and tutorials which form the core of the OHNS curriculum at the University of Sydney. Online e-Learning resources have demonstrated high approval ratings with students, as modern education is shifting from a traditional instructor-centered teaching to a learner-centered model by putting students in control of their own education [5, 23]. Furthermore, online e-Learning resources are designed to improve the ease of access of medical knowledge to all medical students, as they are universally available regardless of geographic location or time limitations [24]. It is anticipated that the role of the e-Learning resources is to enable students to access relevant information before formal OHNS teaching, in order to stimulate a more engaging discussion between students and teachers during classroom-based lectures or tutorials.

To determine the effectiveness of the online e-Learning resources in supplementing OHNS teaching in medical school, OSCE, a competency-based assessment pioneered by Ronald Harden at the University of Dundee, Scotland, was the modality of choice in student assessment. This is because OSCEs are practical to deliver and allow the standardization of assessment conditions for students, facilitating better comparisons between the two groups [25, 26]. Furthermore, the OSCE process is particularly important in the minimization of intra-observer variability in medical school examinations, as a variety of attending physicians (who employ different examination techniques to elicit relevant clinical signs) will have to assess a large volume of students [27].

Results obtained from this pilot study are encouraging – overall and individual OSCE station scores of students in Group A were better than students in Group B, suggesting an improvement in knowledge acquisition and application with additional help from e-Learning resources. Interestingly, students in Group B were able to score better in thyroid and otological examinations compared to the other three clinical examinations. This may be related to the fact that thyroid examination has been covered and practiced in endocrinology syllabus, and otological examination in primary care syllabus.

Following the successful completion of the pilot study, a questionnaire was distributed to better understand the learning needs of each student. We respect student feedback and view this process as ongoing interaction between the students and academic staff to improve the teaching content and method of delivery for medical students. The questionnaire responses showed that students found the e-Learning resources helpful in understanding basic OHNS concepts in a clear manner and beneficial for revision purposes. Furthermore, students were all in agreement that e-Learning is a useful modality in supplementing OHNS teaching, with no clear differences in responses between students in the intervention and the control group, nor students who passed or failed.

The results and the positive student feedback from our pilot study highlight the primary advantage of having freely available interactive e-Learning resources, as students have the flexibility to access the resources at a time that is convenient to achieve their learning goals. Students are able access the resources conveniently, which encourages focused repetition and consolidation of knowledge prior to formal lectures and tutorials provided by the medical school. Similar results have been demonstrated through use of online clinical examination videos to supplement endocrinology teaching in the University of Sydney medical school, providing external validity to our study [28].

Further supporting the utility of the online e-Learning resources in supplementing lecture-based teaching, the literature suggests that online video resources may be a more effective and time-efficient way of delivering educational content compared to other mediums. A study by Buch et al. compared video and illustrated text-based e-Learning in the teaching of Dix-Hallpike maneuver, where students who watched the examination video performed better than those who read the online illustrated textbook for both the primary and follow-up assessments [29]. Similarly, Shippey et al. found that students had improved knowledge retention when a training video was used to supplement face-to-face teaching in subcuticular suturing [30]. In addition, Steedman et al. compared student education on acute eye conditions through video or textbook-based learning, and found that both groups performed equally well on multiple choice assessment despite less time spent studying from the video compared to textbook reading (mean of 8 min v. 29 min respectively; *P* = < 0.01) [7].

This pilot study was limited by its small sample size, giving wide confidence intervals for the differences in OSCE performance between student groups. Having a volunteer group of medical students to participate in this study may lack generalizability, as participating students may not be representative of the entire final-year medical student cohort. There may also be a degree of measurement bias given that there were six examiners who participated in the study, despite prior instructions to ensure marking consistency. It is acknowledged that better results of students in Group A may be associated with the additional teaching time that they were exposed to through the use of the e-Learning resources, while Group B were asked to ‘study as usual’ and not given additional teaching time. In addition, the server hosting the e-Learning resources was not able to track usage of these resources, which meant that the pilot study was unable to quantify the number of use of these resources by students in Group A. Future studies involving larger numbers of medical students are needed to validate these results.

## Conclusion

Medical students who were given access to the online e-Learning resources were able to perform significantly better than those who did not. Results from our study suggests that the use of interactive online e-Learning resources can be a valuable adjunct in supplementing OHNS teaching in medical school, as they are readily accessible and allow flexible on-demand learning. Future larger scale studies assessing the effectiveness of e-Learning resources in OHNS teaching is necessary to allow validation of their usefulness and eventual implementation into the medical school curriculum. This is important to achieve, as basic knowledge of OHNS is an area educational need which should be focused upon in order for students to become safe and competent practitioners in the future.

## Data Availability

The datasets used and analyzed during the current study are available from the corresponding author on reasonable request.

## Abbreviations

OHNS: Otolaryngology Head and Neck Surgery
OSCE: Objective Structured Clinical Examination
